# Silence is golden, but my measures still see—why cheaper-but-noisier outcome measures in large simple trials can be more cost-effective than gold standards

**DOI:** 10.1186/s13063-024-08374-5

**Published:** 2024-08-12

**Authors:** Benjamin Woolf, Hugo Pedder, Henry Rodriguez-Broadbent, Phil Edwards

**Affiliations:** 1 Department of Psychological Science, University of Bristol, 12a Priory Road, Bristol, BS8 1TU UK; 2 Medical Research Council Integrative Epidemiology Unit, University of Bristol, Bristol, UK; 3 Faculty of Epidemiology and Population Health, London, School of Hygiene and Tropical Medicine , London, UK; 4 Medical Research Council Biostatistics Unit, University of Cambridge, Cambridge, UK; 5 Department of Population Health Sciences, Bristol Medical School, University of Bristol, Bristol, UK; 6 Department of Mathematics, Imperial College London, London, UK

**Keywords:** Outcome assessment, Questionnaires, Measurement error, Sampling error, Loss to follow-up, Response rate, Questionnaire length

## Abstract

**Objective:**

To assess the cost-effectiveness of using cheaper-but-noisier outcome measures, such as a short questionnaire, for large simple clinical trials.

**Background:**

To detect associations reliably, trials must avoid bias and random error. To reduce random error, we can increase the size of the trial and increase the accuracy of the outcome measurement process. However, with fixed resources, there is a trade-off between the number of participants a trial can enrol and the amount of information that can be collected on each participant during data collection.

**Methods:**

To consider the effect on measurement error of using outcome scales with varying numbers of categories, we define and calculate the variance from categorisation that would be expected from using a category midpoint; define the analytic conditions under which such a measure is cost-effective; use meta-regression to estimate the impact of participant burden, defined as questionnaire length, on response rates; and develop an interactive web-app to allow researchers to explore the cost-effectiveness of using such a measure under plausible assumptions.

**Results:**

An outcome scale with only a few categories greatly reduced the variance of non-measurement. For example, a scale with five categories reduced the variance of non-measurement by 96% for a uniform distribution. We show that a simple measure will be more cost-effective than a gold-standard measure if the relative increase in variance due to using it is less than the relative increase in cost from the gold standard, assuming it does not introduce bias in the measurement. We found an inverse power law relationship between participant burden and response rates such that a doubling the burden on participants reduces the response rate by around one third. Finally, we created an interactive web-app (https://benjiwoolf.shinyapps.io/cheapbutnoisymeasures/) to allow exploration of when using a cheap-but-noisy measure will be more cost-effective using realistic parameters.

**Conclusion:**

Cheaper-but-noisier questionnaires containing just a few questions can be a cost-effective way of maximising power. However, their use requires a judgement on the trade-off between the potential increase in risk of information bias and the reduction in the potential of selection bias due to the expected higher response rates.

**Supplementary Information:**

The online version contains supplementary material available at10.1186/s13063-024-08374-5.

## Key messages

- A cheaper-but-noisier outcome measure, like a short form questionnaire, is a more cost-effective method of maximising power in large simple clinical trials than an error free gold standard measure when the percentage increase in noise from using the cheaper-but-noisier measure is less than the relative difference in the cost of administering the two measures.
- We have created an R-shiny app to facilitate the exploration of when this condition is met at https://benjiwoolf.shinyapps.io/cheapbutnoisymeasures/
- Cheaper-but-noisier outcome measures are more likely to introduce information bias than a gold standard but may reduce selection bias because they reduce loss-to-follow-up. Researchers therefore need to form a judgement about the relative increase or decrease in bias before using a cheap-but-noisy measure.
- We encourage the development and validation of short form questionnaires to enable the use of high quality cheaper-but-noisier outcome measures in randomised controlled trials.

**Supplementary Information:**

The online version contains supplementary material available at10.1186/s13063-024-08374-5.

## Introduction

### Large simple trials

Many clinical drug trials include at most a few hundred participants [1, 2], who meet a narrow set of criteria and collect large amounts of very specific data on each participant. Recruitment, data collection, monitoring, and auditing processes in these trials are therefore often expensive [3].

Large simple trials (LSTs), on the other hand, include a few thousand participants (to ensure they are sufficiently powered to reliably detect small to moderate intervention effects) and use a simplified design to minimise bias and random error. The simplified design of LSTs usually includes simple randomisation, broad eligibility criteria (leading to a large, diverse patient population and increased generalisability of the study results), a focus on meaningful outcomes important to patient care, and a streamlined approach to collecting data on these outcomes efficiently [3]. Some LSTs are needed to answer important questions reliably [4, 5].

An example of a LST is the International Stroke Trial (IST), a randomised trial of aspirin, subcutaneous heparin, both, or neither, which successfully randomised 19,435 patients with acute ischaemic stroke entering 467 hospitals in 36 countries between March 1993 and May 1996 [6]. In this LST, outcome at 6 months (dependency and incomplete recovery) was assessed using two simple questions asked of the patient, carer, or relative, by questionnaire or telephone interview [6, 7]. There was minimal loss to follow-up using this simple data collection approach (99% follow-up was achieved at 6 months) [6]. Earlier support for simpler health status measures was provided in a study of depression in older adults where a single question was found to be as accurate as the 30 question Geriatric Depression Scale (GDS) [8].

### Simple outcome measures can convey a large amount of information

In support of outcome assessment using simple questions in the IST, Dorman et al. referred to a personal communication from Richard Peto, in which he claimed that ‘simple categorical data of the type generated by simple questions, can convey a large amount of information. For example, if a theoretical highly accurate outcome measurement scale has a possible range of 0 to 100, a simple measure which can identify participants with a score of 0–50 and those scoring 51–100 would reduce the variance of non-measurement by about 75%. In other words, three quarters of the information which would have been obtained using the full 100-point scale (at great effort, time, and cost) can be obtained by a simple dichotomy. An equal three-way split reduces the variance by 89%’ [9].

One approach to simple measurement has been widely adopted by psychologists, where diagnoses are typically based on questionnaire-based measurement instruments rather than clinical judgement [10, 11]. In classical psychometrics, a common way of constructing instruments is by using questionnaires requiring many ‘yes/no’ responses or ‘Likert scales’. Likert scales are traditionally questions with 5 (or 3) levels of response (e.g. ‘strongly agree’, ‘agree’, ‘neutral’, ‘disagree’, ‘strongly disagree’) which are each given a numerical value in analysis (e.g. 1, 2, 3, 4, 5). Although technically an ordered categorical variable, the summation of many Likert questions tends to approximate a normal distribution and is therefore treated as ordinal or ratio scales [12, 13]. ‘Yes/no’ responses are also typically given numerical values of 1 or 0 and summed in the analysis. Computerised questionnaires sometimes avoid this issue by replacing the categorical question with a truly continuous scale. If these instruments are valid, using them as a measure of the continuous underlying liability of the outcome will have greater power than an analysis which dichotomised the trait based on a clinical threshold [14]. For disorders in which a ‘gold standard’ measure, such as a doctor’s diagnosis or biometric measurement is available, these scales can be validated by comparing outcome measurements on the scale with those obtained by using the gold standard [15]. As such, questionnaires can be a simpler approach to measuring outcomes.

### Measurement error

The quantification of measurement error in epidemiology uses the classical (i.e. non-differential) measurement error model, which assumes that the measured value of an outcome, $\widehat{y}$, varies around the true outcome value, *y*, such that $\widehat{y}=y+e$, where the error, *e*, is often assumed to be normally distributed with mean 0 and variance ${\sigma }^{2}$ [16].

Use of simple outcome measures introduces measurement error from categorising participant outcomes on a continuous scale and assuming a common outcome value for all participants within each outcome category: This variance is from differences between true outcome values and the values assigned to the outcome categories, which we define here as the *variance from outcome categorisation* (see the ‘Methods’ section).

For example, the GAD-7 measures Generalised Anxiety Disorder (GAD) using seven 4-level questions which are summed into a 28-level scale. Each question asks participants to rate their symptoms of anxiety over the past 2-weeks: ‘not at all’ (scored as 0), ‘lasting several days’ (scored as 1), ‘lasting over half the days’ (scored as 2), and ‘nearly every day’ (scored as 3). If we assume that there is a continuous underlying liability to anxiety, then this scale is used as a continuous measure of anxiety, or it may be transformed into a binary diagnostic proxy; researchers implicitly assume a homogenous distribution of participants’ actual liability to GAD within each measured group [17].

The *variance of non-measurement* is variance from outcome categorisation using an outcome rating scale with just one category. In other words, when no attempt at measurement is made, every participant is treated equally and is assigned to the midpoint of the scale.

### Trade-off between trial size and data collection burden on participants

When resources are fixed, there is a trade-off between the number of participants a trial can enrol and the amount of information that can be collected on each participant. In LSTs, collecting outcome data by telephone interview, postal, or online questionnaires may be the only financially viable options. For example, if £50,000 was available for outcome data collection and the cost of a detailed assessment of outcome by a trained nurse or doctor was £50 per patient, it would be possible to assess outcomes on 1000 participants. If the cost of sending a validated outcome questionnaire and two reminders to each participant or their carer was £5 per participant, it would be possible to assess outcomes on 10,000 participants.

Increased measurement implies greater participant burden, e.g. the amount of effort required by a participant to respond to a longer questionnaire. Greater participant burden is in turn associated with greater loss-to-follow-up. For example, the odds of a participant providing outcome data are 60% greater (OR = 1.58, 95% CI: 1.40 to 1.78) when using a shorter questionnaire than a longer one [18]. However, shorter questionnaires may produce more measurement error than a gold standard outcome assessment and may not necessarily improve a clinical trial’s power despite a larger study size.

### Aim of the study

Our study is set within the framework of LSTs, and our aim is to contribute to trial design in the specific context of LSTs (i.e. post-approval phase IV studies of widely practicable treatments) [5, 19, 20]. Specifically, we aim to facilitate the design of LSTs through exploring the relative merits of using simpler methods to measure patient outcomes, such as a short questionnaire, which may be ‘cheaper-but-noisier’ when compared to a more complicated gold standard measure. We prove Peto’s claim (above), and we then use his theoretical reduction in the variance of non-measurement from using outcome categories in simulations categorising participant outcomes for uniform and normal distributions, to find a decision rule for when it is more cost-effective to use a simple outcome measure rather than a more complicated gold standard measure.

## Methods

### Calculation of Peto’s reduction in the variance of non-measurement using simple outcome measures for different distributions of outcome

We assume that a trial outcome can theoretically be measured perfectly using a highly accurate ‘gold standard’ outcome measurement scale comprising 100 levels (0 to 99). For example, if the outcome was subjective wellbeing, then the gold standard would classify patients between 0 (lowest possible wellbeing) and 99 (highest possible wellbeing) with each integer between indicating increasingly favourable wellbeing outcomes.

### Variance of non-measurement

**Fig. 1:**
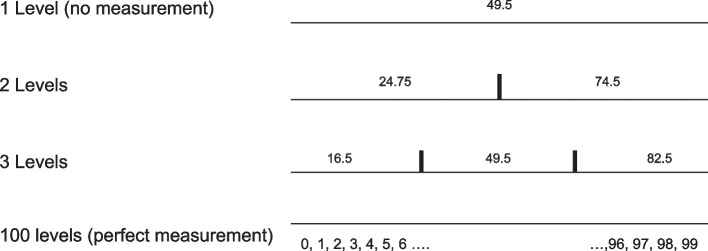
Outcome rating scales that subdivide the entire range of outcomes into 2, 3,… outcome categories

When no attempt at outcome measurement is made, every patient is treated equally and is assigned to the midpoint of the scale. This is equivalent to using an outcome rating scale with just one category. For our analysis, the midpoint of the range of the possible values 0 to 99 is 49.5 (see Fig. 1 and Supplementary Table 1 for more detail).

### Variance from outcome categorisation

One simple question, for example: ‘On a scale between 0 (lowest wellbeing) and 99 (highest wellbeing), do you consider your wellbeing to be below 50?’, will create a dichotomy (2 categories) of outcome. With this simple dichotomy, we assume that the outcomes are the midpoints of the ranges of values covered by each category: 24.75 (midpoint of the range 0 to 49.5, and 74.5 (midpoint of the range 50 to 99). We additionally model scales with 3, 5, 8, 10, and 15 categories and assume that the outcomes are the midpoints of the ranges of values covered by each category.

We define the variance from outcome categorisation (σ_c_^2^) as the variance due to differences between the true outcome value and the midpoint of the scale category to which the outcome is assigned (when a continuous scale is categorised):

$${{\varvec{\upsigma}}}_{{\varvec{c}}}^{2} = \frac{1}{N}\sum_{i=0}^{N}\sum_{m}^{M}{({y}_{i,m}- {\overline{y} }_{m})}^{2}$$

where *y*_*i,m*_ denotes the true outcome value in participant *i* within category *m*, and ${\overline{y} }_{m}$ denotes the midpoint of the range of values in category *m. M* denotes the total number of outcome categories and *N* the total number of participants.

We calculate ${\upsigma }_{c}^{2}$ for measures with 1, 2, 3, 5, 8, 10, or 15 outcome categories using a uniform distribution ranging from 0 to 99 and using constrained discrete normal distributions with a range of 0 to 99, a mean of 49.5, and standard deviations ranging from 0.5 to 25, at 0.5 intervals. As we have said above, ‘no measurement’ is equivalent to having the population take a one category question which assigns everyone to the mid-point value of the scale.

### Reduction in variance of non-measurement from outcome categorisation

The reduction in variance of non-measurement from outcome categorisation is the difference between the variance of non-measurement and the variance from outcome categorisation.

### Analytic conditions for simple measures to be cost-effective

The sample size required to achieve sufficient power in a simple two arm trial is given by the formula: $n=\text{F}({\sigma }_{1}^{2}+{\sigma }_{2}^{2})/{d}^{2}$ [21], where *n* is the sample size in each arm (assuming equal sized groups), $F$ is a function of the critical values of the standard normal distribution for a type I error of $\alpha$ and a type II error of $\beta$, ${\sigma }_{1}$ and ${\sigma }_{2}$ are the standard deviations in each group, and $d$ is the difference in means to be detected. Using this formula and assuming that there is no differential measurement error, we calculate the analytic conditions under which a simple measure will be more cost-effective than a more expensive but less noisy measure, where cost-effectiveness is defined as achieving the same power for a smaller cost.

### Measuring the effect of participant burden

To estimate the effect of increasing participant burden on losses to follow-up, we analysed data from randomised controlled trials of methods to increase response to questionnaires in which participants were randomly assigned to either a longer or shorter questionnaire Edwards et al. [18]. We extracted data from these trials on sample size, odds of response in each arm, risk of bias, and the nature of the questionnaires. Trials which were judged to be at high risk of bias were excluded. We then regressed the logged ratio of the odds of responding on the logged ratio of the length of questionnaires using the meta package in R [22, 23]. Studies were weighted by combining their inverse variance and an additive random effect. Between-study heterogeneity was estimated using a restricted maximum likelihood function.

### Illustrative simulation of when simple measures are more cost-effective and interactive web-app

We used a simulation to explore the comparative effects of using a cheaper-but-noisier measure relative to a gold standard. In our simulation, we model a power of 90%, an alpha of 5%; we additionally chose a low concurrent validity (*r* = 0.7) for the cheaper-but-noisier measure and a small effect size (mean difference = 0.1). Using costs based on the CRASH-1 trial [24], we model the costs of administering the gold standard as £50 and of the cheaper-but-noisier measure as £5 per participant. We then measure the ratio of the costs of measures (with 3, 5, 8, 10, or 15 outcome categories) for a range (0–25) of outcome standard deviations. Consistent with our theoretical ‘gold standard’ outcome measurement scale (above), we assumed the gold standard had 100 levels (0–99) and that the outcome was normally distributed with a mean in the population of 49.5. Because the length of the gold standard was to some extent arbitrary, we ran the simulation with and without any effect of loss to follow-up, additionally assuming that the cost of each lost participant was the same as the cost of a followed-up participant. Graphics were created using GG-Plot [25].

A definitive simulation is difficult because other study-specific factors will influence the equation to decide on which design is more suitable. For example, the validity of the measures, the costs of both measures, the expected size of intervention effect, and the amount of variability of the outcome in the population. In addition, in practice, researchers generally do not measure measurement error using a variance statistic but instead use correlation. Because the parameters we chose may not be the ones of interest in practice, we also created an R-Shiny App to allow researchers to use different parameters to those we have chosen. However, we reiterate that our simulation is primarily for illustrating the theoretical results of our study, rather than to provide guidance on questionnaire constructure for a specific research question.

## Results

### Reduction in variance of non-measurement from outcome categorisation for uniform and normal distributions

The reduction in variance of non-measurement from outcome categorisation for the distributions defined above, using scales with 2, 3, 5, 8 and 10 categories, is shown in Table 1 and Supplementary Table 2. Most of the variance of non-measurement was reduced after adding 3 to 5 categories. For example, for the uniform distribution, the scale with 2 outcome categories resulted in a 75% reduction in variance of non-measurement. Increasing the number of outcome categories to 3 or 5 resulted in 89% or 96% reductions, respectively. The reductions achieved by using scales with larger numbers of outcome categories diminish rapidly (Supplementary Fig. 1). For the normal distributions, the σ_c_^2^ was also reduced by having a scale which was better calibrated to describing the variation in the data (demonstrated by the faster reduction in σ_c_^2^ as the standard deviation increased).

**Table 1:** Examples of variance induced by categorisation for different distributions. Each distribution has a range of 0–99; the normal distributions have a mean of 49.5

Number of categories	Variance from categorising (% reduction in compared to no measurement)					
	Normal distribution, SD = 5	Normal distribution, SD = 10	Normal distribution, SD = 15	Normal distribution, SD = 20	Normal distribution, SD = 25	Uniform distribution
1 (no measurement)	25.08 (0.0%)	100.11 (0.0%)	222.51 (0.0%)	362.63 (0.0%)	479.07 (0.0%)	833.25 (0.0%)
2	444.73 (− 1623.8%)	321.56 (− 221.2%)	247.66 (− 11.3%)	214.26 (40.9%)	202.40 (57.8%)	208.28 (75.0%)
3	24.93 (3.4%)	73.56 (26.5%)	90.75 (59.21%)	97.85 (70.0%)	121.27 (74.7%)	92.73 (88.8%)
5	21.60 (16.3%)	33.04 (67.0%)	33.47 (85.0%)	36.63 (89.9%)	51.89 (89.2%)	33.25 (96.0%)
8	13.69 (46.9%)	13.02 (87.0%)	13.11 (94.1%)	15.29 (95.8%)	26.71 (94.4%)	13.06 (98.4)
10	8.41 (67.4%)	8.33 (91.7%)	8.40 (96.2%)	10.29 (97.2%)	20.54 (95.7%)	8.25 (99.0%)
15	3.74 (85.5%)	4.30 (95.7%)	5.26 (97.6%)	5.61 (98.5%)	5.67 (98.8%)	5.46 (99.3%)
100 (perfect measurement)	0 (100%)	0 (100%)	0 (100%)	0 (100%)	0 (100%)	0 (100.0%)

### Analytic conditions under which simpler measures are more cost-effective

The derivation of the analytic conditions under which a simple measure is more cost-effective than a theoretical ‘gold standard’ outcome measurement, assuming equivalent measurement accuracy, can be found in Supplementary Table 3. This demonstrates that if the relative increase in cost from using the expensive measure is greater than the percentage increase in variance from using the simple measure, the simple measure will be more cost-effective.

### The impact of participant burden on non-response

Edwards et al. [18] included 72 trials in their meta-analysis of the impact on questionnaire length on the odds of participants responding. Of these, two trials provided insufficient information to ascertain either the ratio or actual length of questionnaire in each arm, and 14 trials were at high risk of bias. Of the remainder, 42 trials measured the length of questionnaire using the number of pages, 1 trial used the number of questions, 2 trials provided word counts, and 1 trial provided the time needed to complete the questionnaire (Supplementary Table 4). The questionnaires included in the meta-analysis covered a wide range of topics.

The linear meta-regression is presented in Supplementary Fig. 2 and explained 25.4% of the variance in the odds of participants responding. This showed that for every increase of 1 in the log ratio of the length of questionnaire, the log odds ratio for responding increased by − 0.594 (95% CI − 0.894 to − 0.293, SE = 0.153, *p* < 0.001). There was no evidence that the intercept was different from zero (beta = 0.092, SE = 0.168, *p* = 0.586). We additionally did not find any evidence that adding a quadratic term improved model fit (new *r*^2^ = 23.6%). Converting these parameters from the log–log scale to the natural scale, we therefore find a power law relationship: the ratio in OR = RQ^−0.594^, where OR is the ratio in the odds of responding to the longer questionnaire relative to the shorter questionnaire, and RQ is the ratio in questionnaire length of long questionnaire to the short questionnaire. This implies that doubling the length of a questionnaire would reduce response by around one third, and therefore require asking around 50% more people to participate to achieve the same number of outcome observations (Supplementary Fig. 3).

### Simulation and interactive web-app

**Fig. 2:**
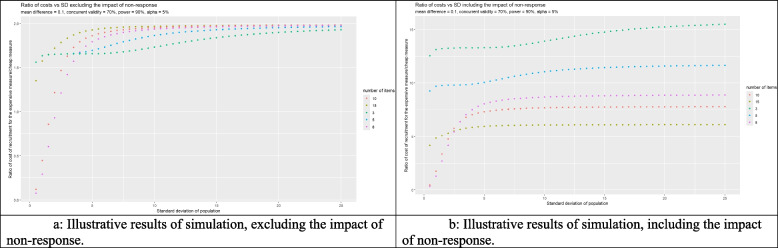
**a** Illustrative results of simulation, excluding the impact of non-response. **b** Illustrative results of simulation, including the impact of non-response

Our simulation found that most cheaper-but-noisier measures outperformed the gold standard when the standard deviation in the population was approximately greater than 1 (Fig. 2). Ignoring the effect of participant burden means that the longer questionnaires, with lower variance from outcome categorisation, performed better than both short questionnaires and the gold standard (Fig. 2a). However, including an effect for loss to follow-up resulted in shorter questionnaires outperforming longer ones and the gold standard (Fig. 2b). We additionally created an R-shiny app (available at https://benjiwoolf.shinyapps.io/cheapbutnoisymeasures/) to allow readers to further explore our simulation using different parameters.

## Discussion

### Principal findings

We have shown that cheaper-but-noisier measures, such as short questionnaires, may be more cost-effective than their gold standard counterparts. Specifically, they will be more cost-effective if the relative increase in cost is greater than the relative increase in variance from measurement error and from outcome categorisation, providing the cheaper-but-noisier measure is valid when compared with the gold standard. This could be achieved by using well-validated short form questionnaires.

We introduced the concept of variance from outcome categorisation (${\upsigma }_{c}^{2}$) and confirmed that even a very simple measure, such as five ‘yes/no’ questions or one five-level Likert scale, can eliminate the vast majority of the variance of non-measurement. ${\upsigma }_{c}^{2}$ was also reduced for normally distributed variables when the scale was calibrated to the variance expected within the population, with more homogeneous populations needing more sensitive scales. This shows the importance of using a measure calibrated to the study population.

Our simulation found that the utility of a simple questionnaire, with few items or response categories, is inversely related to the required sensitivity of the test. If the measure is unable to detect effect sizes as large (or small) as they are expected to be, then the cheap measure is guaranteed to be less useful than a more sensitive measure, even if the gold standard is substantially more expensive. This is analogous to the final part of Smeden et al.’s Triple Whammy of Measurement error, that it can mask features of the data such as effect modification and non-linear associations [26]. Although this is an unsurprising finding, it raises an important caveat that the most appropriate measure will vary depending on factors unique to every trial and that our results do not warrant the use of cheaper-but-noisier measures in every circumstance.

For many trials, there is a cost associated with loss to follow-up. For example, in most trials, there are costs associated with administering the intervention and data collection. An important implication of the association between participant burden (e.g. questionnaire length) and non-response is that, in clinical trials with a cost of non-response, the analytic solution for when a simple measure is more cost-effective will under-estimate the saving associated with using a simpler measure because of the expected higher follow-up. In a meta-regression of a previously conducted Cochrane systematic review, we found that the ratio of response rates has an inverse power law relationship with questionnaire length, such that doubling the participant burden (measured as questionnaire length) will reduce the response rate by around one third.

### Simple measures and risk of selection bias

One source of bias is due to differential response (selection bias). As our study shows, simpler questionnaires have meaningfully lower loss to follow-up. This implies that an additional advantage of the use of cheaper-but-noisier measures is a reduction in the risk of selection bias. For example, the International Stroke Trial assessed disability after stroke for 19,435 participants [6]. Conventional outcome measures (e.g. the Barthel Index and Oxford Handicap Scale) were considered to be too complicated and expensive. Instead, two simple questions with a reasonable validity relative to the Barthel Index and the Oxford Handicap Scale were used to measure handicap [7, 9]. This allowed participants to be classified into three levels: needing help (‘dependent’), not needing help but still with some handicap (‘independent’), and those not needing help and with no handicap (‘independent and recovered’). At follow-up, 6 months after randomisation, the trial achieved a 99% response rate and evidence of a clinically important treatment effect. Even if all data was missing not at random, the potential for serious selection bias is minimal. It is likely that so few losses to follow-up may have been influenced by the decision to use a simple outcome measure.

### Limitations of the study

Our study did not consider all types of measurement error, for example calibration error (which occurs when assigning incorrect units to a scale) and parallax error [27]. These can both be removed by standardising the measure, but more complex errors might be more difficult to address statistically and thus limit the generalisability of our findings to all cheaper-but-noisier measures. Similarly, the results of the meta-regression also may also not be generalisable. Because we also assume a certain level of validity in the cheaper-but-noisier measures, our results do not warrant the use of unvalidated measures. A possible alternative to questionaries, not considered here, is to use linkage to electronic health record data as a cheap and possibly noisy means of outcome data collection.

Finally, although our results are appropriate for outcome collection for clinical trials, they may not be applicable in other settings. For example, exposure-related measurement error will introduce bias, especially for multivariable analysis [26]. Various sensitivity analyses, such as regression calibration and SIMEX, have been developed to attenuate bias due to exposure-related measurement error [28]. However, the extent to which these methods may be useful in overcoming any bias due to variance from outcome categorisation remains unclear. More generally, measurement error is a greater issue for studies attempting to estimate the effect between two well defined phenotypes, than studies simply testing for clinically relevant differences between intervention and control arms. Together, this implies that using cheaper-but-noisier measures in observational studies or mediation analyses could result in misleading findings and should therefore be avoided.

### Selection bias—information bias trade off

Our study has explored the benefits of simple measures in terms of the power of a clinical trial. However, an important limitation is that simple measures may increase risk of information bias due to differential measurement error. Differential measurement error occurs when the error is not random but depends on some other factor related to the patient [29, 30]. Risk of bias is arguably more important than improving power because random error can be eliminated in a meta-analysis of many small studies, while bias cannot. Simple measures, like a questionnaire, may be more suspectable to bias than a more expensive measure for two reasons.

Firstly, a simple questionnaire can be influenced by social and psychological factors for which a more expensive objective measure, like a biometric reading, will not. For example, a researcher may subtly change the way they ask a question about alcohol consumption based of their perception of a participant (i.e. ‘interviewer bias’), while a participant’s knowledge of social expectations may lead them to downplay how much alcohol they have drunk (i.e. ‘social desirability bias’). On the other hand, neither the researcher’s nor participant’s expectations will influence a breathalyser reading. A well-designed questionnaire should be able to eliminate these types of information bias. For example, psychometricians can use methods such as control questions, reverse coding, and an independent rater. A limitation of these methods is that they increase the complexity of outcome measures and therefore may undermine both the simplicity and cost of the cheaper-but-noisier measures. This source of bias can also be attenuated by improving the study design. Blinding can be used to reduce the possibility that any bias is differential across exposure status. Likewise, the use of anonymised postal or online questionnaires may reduce perceived social pressures or other (interviewer) bias due to having study personnel requesting information from participants in-person.

A second source of differential measurement error could arise from the measure itself. Many outcomes are intrinsically complex or multi-dimensional, obvious examples being socio-economic position or frailty. A risk of using a simple measure is that it may not capture all of the desired dimensions of the outcome of interest. When this occurs, it is likely that the simpler measure will produce incorrect estimates of effect. For example, frailty is often thought to involve both physical and psychological dimensions [31]. An evaluation of an intervention designed to reduce frailty may produce misleading results if it only measures the psychological impact of frailty or potentially miss the entire effect if the intervention’s impact is mostly mediated by reducing physical frailty.

Because these sources of information bias are intrinsic to the simplification process, they are a limiting factor on the utility of cheaper-but-noisier measures. The higher participant burden in most gold-standard measures, however, increases risk of selection bias in trials that use them when compared to a cheaper-but-noisier alternative. This implies the existence of a second quality-quantity trade off not explored in our simulation. The amount of selection and information bias in a trial will vary depending on each study’s methods and, if measurable, can only be quantified post-hoc. It is therefore impossible to provide universally applicable prescriptions beyond attempting to minimise the overall risk of bias in a trial. With this in mind, we believe that cheaper-but-noisier measures should be considered with caution in trials where information bias is likely but may be useful in reducing the overall risk of bias in trials with a greater risk of selection bias than information bias.

## Conclusion and implications for outcome measures in large simple clinical trials

Large simple trials will become more cost-effective by employing cheaper-but-noisier outcome measures, such as a simple questionnaire, when the relative increase in cost between the cheaper-but-noisier measure and its gold standard alternative is greater than the relative increase in variance, assuming no bias. Simple questionnaires, with a given level of validity, have the added advantages of reducing loss to follow-up by improving response rates and not adding large amounts of noise. However, the relative merits of doing so will vary from study to study. Importantly, any increase in power and reduction of susceptibility to selection bias must be balanced against a potential increase in information bias. Table 2 provides a checklist of questions we hope will provide readers with a useful screen for when not to use a cheap-but-noisy measure. Finally, we have assumed throughout the existence of a previously created, and validated, questionnaire that could be used as a cheaper-but-noisier outcome measure. Although questionnaires are becoming increasingly popular as health measures, see for example references [32–35], we would encourage the development and validation of a wider range of questionnaires to enable their use as endpoints in clinical trials. As per ICH-GCP(R3), these endpoints should measure meaningful trial outcomes, supported by the perspectives of stakeholders (e.g. patients and/or healthcare providers) [36].

**Table 2:** Checklist for screening cheap-but-noisy measures

1. Is there a candidate cheap but noisy measure?
2. What are the units of the measure? If it is not standardised, is there a risk of calibration error or a non-differential bias like parallax error?
3. Does the cheap-but-noisy measure have sufficient sensitivity to detect the expected effect and not mask any important variation or features of the data?
4. Is the outcome simple or multidimensional? If multidimensional, does the cheap-but-noisy measure capture signal from all relevant dimensions?
5. Is there a material risk of response biases like interviewer or recall bias?
6. Are there changes to the measure design (such as control questions, reverse coding, independent-rater, etc.) or study design (e.g. blinding of participants and study personnel, online questionnaires, etc.) that could attenuate a response bias?
7. Biased off question 4. to 6. what is the likely overall size and direction of any information bias that using a cheap-but-noisy measure could introduce?
8. Is there an expected increase in sample size from using a cheap-but-noisy measure? What is the expected size and direction of a reduction in risk of selection bias?
9. Does the reduction in risk of selection bias outweigh any increase in the risk of information bias?
10. Has a cheap-but-noisy measure been validated? If not, we should suggest authors conduct a validation study if possible
11. How much less expensive is the Is the cheap-but-noisy measure?
12. How much more noisy is the cheap-but-noisy measure?
13. Howe reliable is the estimation of the above two numbers?
14. Is the cheap-but-noisy measure cost-effective?

### Supplementary Information

Supplementary Material 1: Supplementary Fig. 1. Percentage reduction in variance from categorisation from adding measurement levels, compared to no measurement

Supplementary Material 2: Supplementary Fig. 2. Results of the meta-regression, with both variables presented on the log–log scale. Both ratios are defined as the ratio in length of long questionnaire to short questionnaire

Supplementary Material 3: Supplementary Fig. 3. Illustration of the effect of increasing questionnaire length on reducing response rates from meta-regression. The y-axis can be interpreted as the multiple of how many more participants would be needed to achieve the same number of responses given how many times larger the questionnaire being used is

Supplementary Material 4: Supplementary Table 1. Ranges and values of categories for questionnaire of different length

Supplementary Material 5: Supplementary Table 2. The reduction in variance of non-measurement from outcome categorisation for the distributions defined using scales with 2, 3, 5, 8 and 10 categories

Supplementary Material 6: Supplementary Table 3. Analytic conditions for when a simple measure is more cost-effective

Supplementary Material 7: Supplementary Table 4. Comparison of final response

## Data Availability

The R code used to calculate the variance from categorisation and run the simulation can be found at https://github.com/bar-woolf/applied-MR-code/blob/main/Silence%20is%20golden%20code.R.
