# Supplementary material for: Silence is golden, but my measures still see—why cheaper-but-noisier outcome measures in large simple trials can be more cost-effective than gold standards
Source: Trials. 2024 Aug 12;25:532. doi: 10.1186/s13063-024-08374-5 (PMC11318131; doi:10.1186/s13063-024-08374-5)
Supplement: Supplementary file 1 — Supplementary Material 1: Supplementary Fig. 1. Percentage reduction in variance from categorisation from adding measurement levels, compared to no measurement [file 13063_2024_8374_MOESM1_ESM.docx]

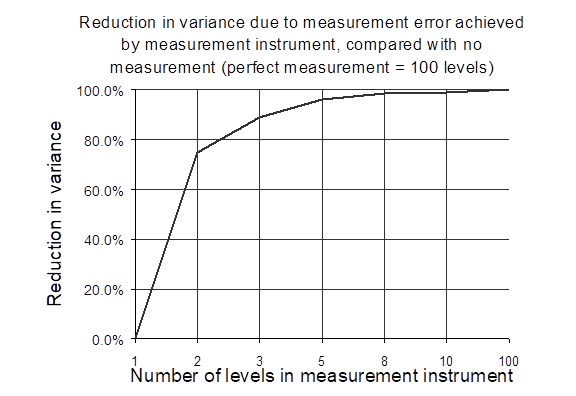


Supplementary Figure 1: Percentage reduction in variance from categorisation from adding measurement levels, compared to no measurement.
