# Supplementary material for: Silence is golden, but my measures still see—why cheaper-but-noisier outcome measures in large simple trials can be more cost-effective than gold standards
Source: Trials. 2024 Aug 12;25:532. doi: 10.1186/s13063-024-08374-5 (PMC11318131; doi:10.1186/s13063-024-08374-5)
Supplement: Supplementary file 2 — Supplementary Material 2: Supplementary Fig. 2. Results of the meta-regression, with both variables presented on the log–log scale. Both ratios are defined as the ratio in length of long questionnaire to short questionnaire [file 13063_2024_8374_MOESM2_ESM.docx]

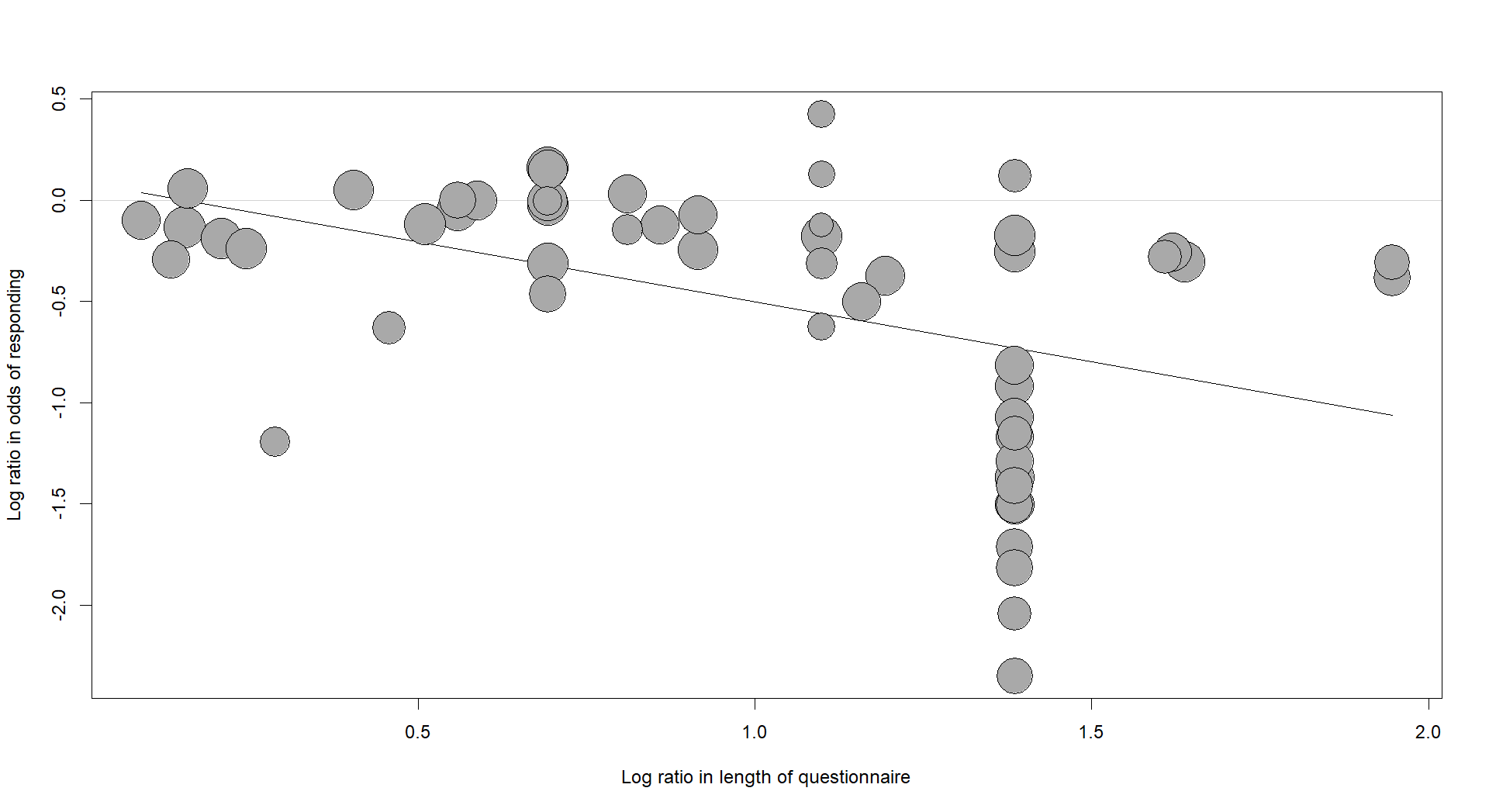


Supplementary Figure 2: Results of the meta-regression, with both variables presented on the log-log scale. Both ratios are defined as the ratio in length of long questionnaire to short questionnaire.
