# Supplementary material for: Silence is golden, but my measures still see—why cheaper-but-noisier outcome measures in large simple trials can be more cost-effective than gold standards
Source: Trials. 2024 Aug 12;25:532. doi: 10.1186/s13063-024-08374-5 (PMC11318131; doi:10.1186/s13063-024-08374-5)
Supplement: Supplementary file 3 — Supplementary Material 3: Supplementary Fig. 3. Illustration of the effect of increasing questionnaire length on reducing response rates from meta-regression. The y-axis can be interpreted as the multiple of how many more participants would be needed to achieve the same number of responses given how many times larger the questionnaire being used is [file 13063_2024_8374_MOESM3_ESM.docx]

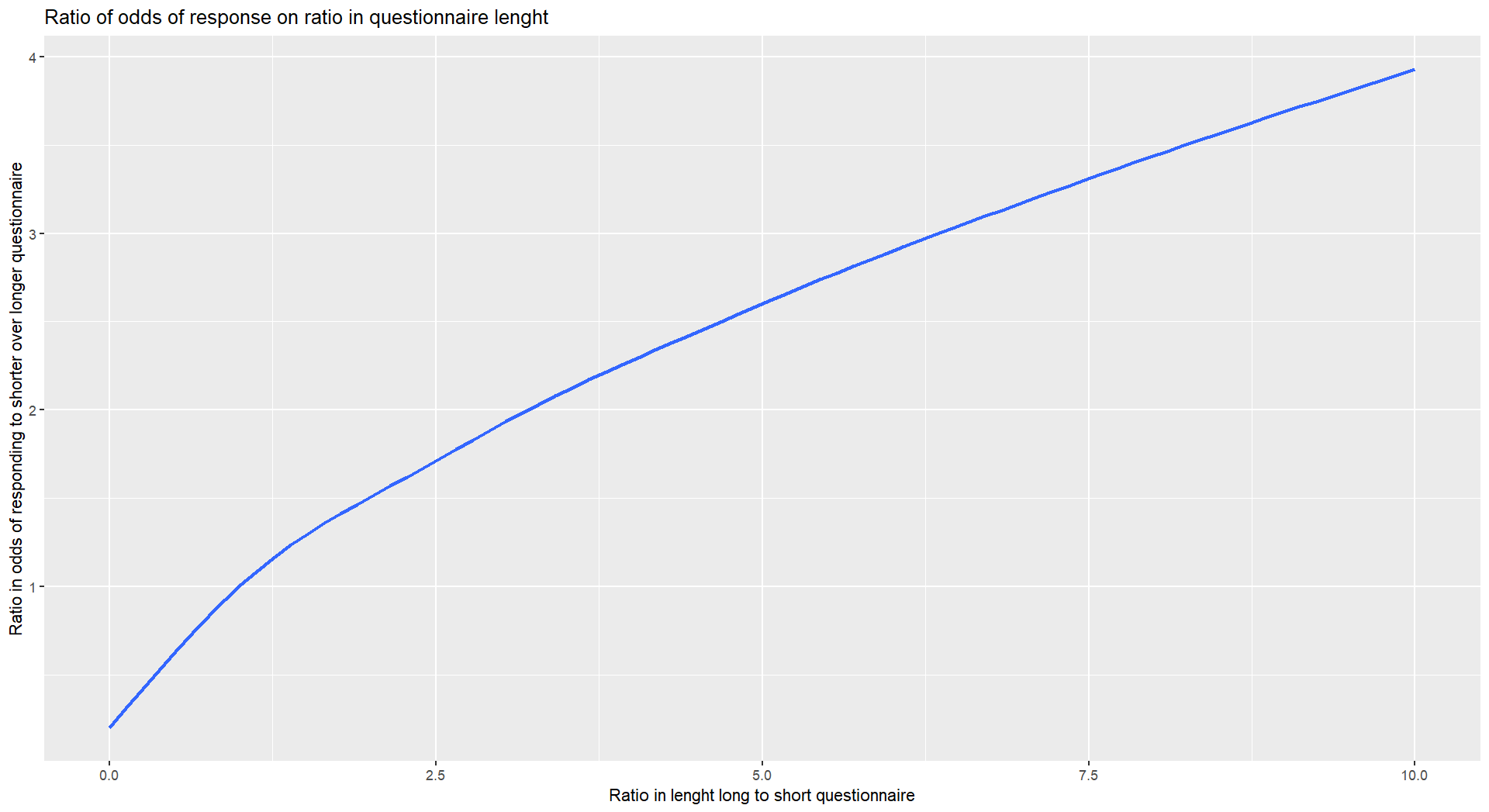


Supplementary Figure 3: Illustration of the effect of increasing questionnaire length on reducing response rates from meta-regression. The y-axis can be interpreted as the multiple of how many more participants would be needed to achieve the same number of responses given how many times larger the questionnaire being used is.
